# Focus on Pivotal Role of Dietary Intake (Diet and Supplement) and Blood Levels of Tocopherols and Tocotrienols in Obtaining Successful Aging

**DOI:** 10.3390/ijms161023227

**Published:** 2015-09-25

**Authors:** Mariangela Rondanelli, Milena Anna Faliva, Gabriella Peroni, Francesca Moncaglieri, Vittoria Infantino, Maurizio Naso, Simone Perna

**Affiliations:** 1 Department of Public Health, Experimental and Forensic Medicine, School of Medicine, Endocrinology and Nutrition Unit, University of Pavia, Azienda di Servizi alla Persona di Pavia, Pavia 27100, Italy; E-Mails: mariangela.rondanelli@unipv.it (M.R.); milo.milena87@hotmail.it (M.A.F.); gabriella.peroni01@universitadipavia.it (G.P.); francesca.moncaglieri01@universitadipavia.it (F.M.); viriainfantino@hotmail.it (V.I.); 2 Faculty of Medicine and Surgery, Department of Clinical Sciences, University of Milano, Milan 20100, Italy; E-Mail: maurizio.naso@unimi.it

**Keywords:** tocopherols, tocotrienols, dietary supplement, aging, sarcopenia, osteoporosis, mild cognitive impairment, dementia, vitamin E

## Abstract

Numerous specific age-related morbidities have been correlated with low intake and serum levels of tocopherols and tocotrienols. We performed a review in order to evaluate the extant evidence regarding: (1) the association between intake and serum levels of tocopherols and tocotrienols and age-related pathologies (osteoporosis, sarcopenia and cognitive impairment); and (2) the optimum diet therapy or supplementation with tocopherols and tocotrienols for the treatment of these abnormalities. This review included 51 eligible studies. The recent literature underlines that, given the detrimental effect of low intake and serum levels of tocopherols and tocotrienols on bone, muscle mass, and cognitive function, a change in the lifestyle must be the cornerstone in the prevention of these specific age-related pathologies related to vitamin E-deficient status. The optimum diet therapy in the elderly for avoiding vitamin E deficiency and its negative correlates, such as high inflammation and oxidation, must aim at achieving specific nutritional goals. These goals must be reached through: accession of the elderly subjects to specific personalized dietary programs aimed at achieving and/or maintaining body weight (avoid malnutrition); increase their intake of food rich in vitamin E, such as derivatives of oily seeds (in particular wheat germ oil), olive oil, hazelnuts, walnuts, almonds, and cereals rich in vitamin E (such as specific rice cultivar rich in tocotrienols) or take vitamin E supplements. In this case, vitamin E can be correctly used in a personalized way either for the outcome from the pathology or to achieve healthy aging and longevity without any adverse effects.

## 1. Introduction

Suboptimal micronutrient intake for particular vitamins and between these, vitamin E is common in older adults [1] due to both acute conditions and chronic diseases very frequent in the elderly population.

Various factors contribute to this nutritional deficiency in aging with subsequent chronic inflammation, immune response impaired, and increased antioxidant activity. Among them, malnutrition and the intestinal malabsorption are the more common causes of an inadequate vitamin and nutritional support in elderly [2].

Numerous studies showed that inadequate vitamin E intake was highly prevalent in the free living American elderly population, with only 8%–11% of men and 2%–8% of women meeting the estimated average requirement (EAR) for vitamin E from foods alone in the 1994–1996 Continuing Survey of Food Intakes by Individuals (CSFII) [3] and the 2001–2002 National Health and Nutrition Examination Survey (NHANES) [4]. Moreover, the same results have been shown in a representative sample of Puerto Rican and Dominican free living elders and neighborhood-matched non-Hispanic white elders living in Massachusetts: most (94% and 95%, respectively) did not meet the EAR for vitamin E from food alone and fewer than 10% of subjects in both groups had plasma α-tocopherol concentrations <16 mmol/L in both sex [4].

In addition, further studies conducted over the last 10 years in the United States have shown that the percentage of the population over the age of 71 years eating levels below the EAR is equal to 75% for vitamin E [5].

Finally, a clear decreasing trend with age was observed for intake and serum vitamin E and for the α-tocopherol/cholesterol ratio in a group of institutionalized older populations [6] and in hospitalized elderly patients [7]. In the study by Granado-Lorencio, it has been demonstrated that high levels of C-Reactive Protein (CRP) and ferritin, two significant markers of inflammation, were associated with lower serum levels of vitamin E. Consequently, in hospitalized subjects, an inadequate nutritional status and a high inflammation milieu were associated with a higher prevalence of vitamin E deficiency (up to 15%).

Even the National Dietary Survey 2008–2009, which involved more than 4000 elderly people in Brazil, has shown that the prevalence of inadequate intake of several micronutrients, including vitamin E, reaches about 80% in both sexes [8]. However, this deficiency in vitamin E intake can be corrected by improving the diet of the subjects: in healthy elderly residents in the community a more balanced diet (including a good supply of fruit and vegetables) has led to an increase in the share of vitamins [9].

Intake of α-tocopherol below 12 mg/d has been shown to relate to a risk of hydrogen peroxide-induced hemolysis [10], and there is evidence of association with several major chronic diseases, including Alzheimer’s disease, diabetes, cancer, and cardiovascular disease [11,12,13,14].

On the other hand, the presence of good circulating levels of vitamin E in centenarians coupled with satisfactory antioxidant activity and immune response [15], clearly testify the relevance of vitamin E in the economy of the immune and antioxidant performances required to achieve healthy aging and longevity.

Free radicals and oxidative stress have been recognized as important factors in the biology of aging and in many age-associated degenerative diseases [16]. A time-dependent shift in the antioxidant/pro-oxidant balance, which leads to higher free radical generation, increased in oxidative stress and dysregulation of cellular function, is the basis for the free radical theory of aging [17].

Vitamin E is considered the major chain-breaking antioxidant in plasma, in cell membranes and in tissues [18], capable of reacting directly with chain-carrying radicals and consequently interrupt the oxidative chain reactions [19,20]. Tocopherol serves as a peroxyl radical scavenger that protects polyunsaturated fatty acids in membranes and lipoproteins [21]. Apart from its antioxidant property, vitamin E has been reported to also enhance immune response [22] and to modulate DNA repair systems [23] and signal transduction pathways [24].

Given this background, the aim of the present narrative review is to evaluate the till-now evidence regarding: (1) the association between intake and serum levels of tocopherols and tocotrienols and specific age-related pathologies (osteoporosis, sarcopenia, and cognitive decline); and (2) the optimum diet therapy or supplementation with tocopherols and tocotrienols for the treatment of these abnormalities.

## 2. Methods

**Figure 1:**
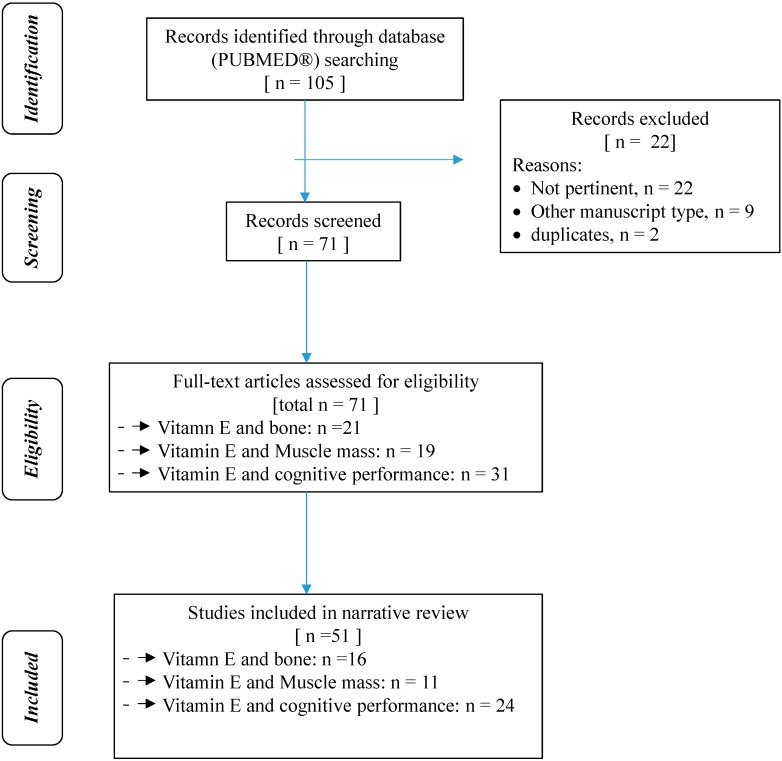
Flow diagram of narrative review of literature.

The present systematic review was performed following the steps by Egger *et al.* as follows [25]: (1) configuration of a working group: three operators skilled in clinical nutrition, of whom one acting as a methodological operator and two participating as clinical operators; (2) formulation of the revision question on the basis of considerations made in the abstract: “the state of the art on metabolic and nutritional correlates (osteoporosis, sarcopenia, cognitive decline) of vitamin E deficiency in elderly and their nutritional treatment”; (3) identification of relevant studies: A research strategy was planned, on PubMed (Public MedIine run by the National Center of Biotechnology Information (NCBI) of the National Library of Medicine of Bathesda (USA)), as follows: (a) definition of the key words (vitamin E, tocopherols, tocotrienols, osteoporosis, bone mass, sarcopenia, muscle mass, Alzheimer’s disease, and mild cognitive impairment), allowing the definition of the interest field of the documents to be searched, grouped in inverted commas (“…”) and used separately or in combination; (b) use of: the boolean (a data type with only two possible values: true or false) AND operator, that allows the establishments of logical relations among concepts; (c) research modalities: advanced search; (d) limits: time limits: papers published in the last 20 years; humans; languages: English; and (e) manual search performed by the senior researchers experienced in clinical nutrition through the revision of reviews and individual articles on vitamin E and specific age-related pathologies (osteoporosis, bone mineral density, sarcopenia, muscle mass, Alzheimer’s disease, mild cognitive impairment, cognitive performance) in the elderly published in journals qualified in the Index Medicus; and (4) analysis and presentation of the outcomes: the data extrapolated from the revised studies were collocated in tables; in particular, for each study we specified: the author and year of publication of the study, study characteristics (type of study, subjects studied, primary end-point), and the main results of the study. The analysis was carried out in the form of a narrative review of the reports. Table 1 summarizes the methodology of the review. At the beginning of each section, the keywords considered and the kind of studies chosen has been reported. Suitable for the systematic review were human studies of any design, which considered the elderly (over 65 years) and the evaluation of food/supplement intake and/or blood levels of congeners of vitamin E. *In vitro* or animal studies are given in the paper only if useful to better explain a result in humans, but are not considered in the tables. Figure 1 shows the flow diagram of the narrative review.

**Table 1:** Summary of methodology.

Step	General Activities	Specific Activities
Step 1	configuration of a working group	Three operators skilled in clinical nutrition: one operator acting as a methodological operator two participating as clinical operators
Step 2	formulation of the revision question	Evaluation of the state of the art on metabolic and nutritional correlates (osteoporosis, bone mineral density, sarcopenia, muscle mass, Alzheimerʼs disease, mild cognitive impairment, cognitive performance) of vitamin E deficiency in elderly and their nutritional treatment
Step 3	identification of relevant studies on Pub Med	Suitable for the systematic review were human studies of any design, which considered elderly (over 65 years). In vitro or animals studies are given in the paper only if useful to better explain a result in humans, but are not considered in the tables
Step 4	analysis and presentation of the outcomes	The data extrapolated from the revised studies was carried out in the form of a narrative review of the reports and were collocated in tables

## 3. Vitamin E and Bone Health

This research has been carried out based on the keywords: “vitamin E” or “tocopherols” or “tocotrienols” AND “osteoporosis” AND “bone mineral density”; 16 articles were sourced. Among them, two case control, five observational studies, one randomized control trial, one meta-analysis, five reviews and two animal studies have been selected and discussed.

Human studies on the effect of vitamin E in relation to bone health are still few, but the interest in this field has increasing because of suggestions of a possible protective effect of antioxidants, such as tocopherols and tocotrienols, on bone health [26,27].

During the past decade, it has become evident that the increase in oxidative stress with aging is a fundamental pathogenetic mechanism of age-related bone loss [28] and, also, possibly sarcopenia [29,30]; two important determinants that contribute to the risk of fracture [31,32,33].

The vitamin E component is a potent scavenger of free radicals, and it has been postulated that this vitamin may favorably influence bone and muscle mass due to its antioxidant properties [28,29,34,35].

In addition, antioxidants may also have the potential to affect bone through creating an alkaline environment, reducing urinary calcium excretion, and providing bioactive components [36].

Observational data published very recently in the literature indicate that vitamin E insufficiency is associated with higher fracture risk [37]. In this research two cohort studies, the Swedish Mammography Cohort (SMC; *n* = 61,433 women) and the Uppsala Longitudinal Study of Adult Men (ULSAM; *n* = 1138 men), were used in order to assess vitamin E intake and to correlate this intake with bone mineral density (BMD), evaluating also by fracture risk [37]. During 19 years of follow-up, 14,738 women in the SMC experienced a first fracture at any site (3871 hip fractures). A higher hip fracture rate was observed with lower intakes of α-tocopherol. This study demonstrated that for each 3-mg decrease in α-tocopherol intake, the BMD of the proximal femur, after multivariable adjustment, decreased by 1.1% (95% CI: 0.3, 1.8; *p* = 0.005), the lumbar spine (L1–L4) by 0.8% (95% CI: 20.1, 1.8; *p* = 0.09), appendicular lean mass by 0.8% (95% CI: 0.2, 1.4; *p* = 0.01), and skeletal mass muscle index (SMI) by 0.6% (95% CI: 0.05, 1.18; *p* = 0.03). In addition, for every 3-mg decrease in α-tocopherol intake, the multivariable adjusted OR of osteoporosis at the lumbar spine was 1.20 (95% CI: 1.03, 1.41) and at the proximal femur was 1.46 (95% CI: 1.19, 1.78).

The results of this study are in agreement with a large community-based prospective case-cohort study of elderly men and women in Norway, in which it has been found an increased risk of hip fracture at low serum concentrations of α-tocopherol, which persisted after controlling for potential confounders, including smoking, BMI, physical inactivity, education, self-rated health, vitamin D status, and hours since last meal [38]. There was a 51% (CI 17%–95%) increased risk in the lowest compared to the highest quartiles of α-tocopherol which was attenuated to 37% (CI 5%–77%) when α-tocopherol/total cholesterol ratio was the exposure considered.

Moreover, in previous cross-sectional studies, hip fracture patients had low vitamin E concentrations compared with control subjects at the time of the fracture event [39], and higher post-operative vitamin E concentrations were associated with lower concentrations of inflammatory markers [40]. Finally, high serum concentrations of vitamin E were related to better physical function after the hip fracture [41].

As regards dietary supplementation, results of a small pilot study in 34 women in Canada suggested that supplements of α-tocopherol (600 mg/day) and vitamin C (1000 mg/day) taken for six months could reduce spinal bone loss in aging [26]. In an observational study of 533 community-dwelling non-smoking postmenopausal women in Australia, the duration of use of antioxidant supplements, including vitamins C and E, was inversely associated with the bone resorption marker C terminal telopeptide in serum but not with whole body BMD [27].

Table 2 summarizes the human studies performed to investigating the influence of vitamin E intake or supplementation on bone.

**Table 2:** Influence of vitamin E on bone.

Author	Type of Study	Subjects Studied	Primary Endpoint	Main Results of the Study
Michaëlsson, K., 2014 [37]	Two cohort studies: The Swedish Mammography Cohort (SMC) and the Uppsala Longitudinal Study of Adult Men (ULSAM)	(SMC; *n* = 61,433 women); (ULSAM; *n* = 1138 men)	To determine whether α-tocopherol intake or serum concentration are associated with fracture risk in older women and men.	Low intakes and low serum concentrations of α-tocopherol are associated with an increased rate of fracture in elderly women and men.
Holvik, K., 2014 [38]	Case-cohort study	21,774 men and women aged 65–79 years who participated in four community-based health studies in Norway 1994–2001	To investigate the association between serum α-tocopherol concentrations and risk of hip fracture during up to 11 years of follow-up.	Low serum concentrations of α-tocopherol were associated with increased risk of hip fracture in older Norwegians.
D’Adamo, C.R., 2011 [39]	Observational study	148 women with hip fracture, from the fourth cohort of the Baltimore Hip Studies (BHS4)	To examine how serum α tocopherol and γ-tocopherol concentrations change throughout the year after hip fracture.	Highly cognitively and physically functioning hip fracture patients demonstrated higher vitamin E concentrations.
D’Adamo, C.R., 2012 [40]	Observational study	148 hip fracture patients	To assess whether post-fracture concentration of vitamin E and the carotenoids were associated with lower levels of IL-6 and the soluble receptor of TNF-α.	Higher post-fracture concentrations of vitamin E and the carotenoids were associated with lower levels of inflammatory markers.
D’Adamo, C.R., 2011 [41]	Observational study	148 female hip fracture patients from the Baltimore Hip Studies cohort 4 (BHS4)	To assess the association between serum concentrations of vitamin E with decline in phisical function among older adults.	Serum concentrations of both α- and γ-tocopherol were associated with better physical function after hip fracture.
Dietary Supplemtation								
Chuin, A., 2009 [26]	Randomized controlled trial	34 postmenopausal women randomized in 4 groups (placebo, *n* = 7; antioxidants, *n* = 8; exercise and placebo, *n* = 11; exercise and antioxidants, *n* = 8)	To evaluate the effects of antioxidants supplementation (α-tocopherol 600 mg/die and vit C 1000 mg/die) combined to resistance training on BMD in healthy elderly women.	Significant decrease in the placebo group for lumbar spine BMD, while it remained stable in all other groups. No changes were observed for femoral neck BMD.
Pasco, J.A., 2006 [27]	observational study	533 community-dwelling non-smoking postmenopausal women (subjects were described as antioxidant supplement users if they were current users of vitamins C and/or E at the time of the assessment)	To assess the association among the use of antioxidant supplements, (vitamins C and E) and serum levels of biochemical markers of bone turnover.	The use of antioxidant supplements was inversely associated with the bone resorption marker C terminal telopeptide in serum, but not with whole body BMD.

In conclusion, all studies performed to date have been showed that both high intake and serum concentrations of α-tocopherol are associated with reduced risk of many common sequelae of hip fracture, including decreased physical function, incident frailty, sarcopenia, bone loss, so an adequate vitamin E intake is crucial in order to maintain bone health in elderly subjects because for each 3-mg decrease in α-tocopherol intake, the BMD of the proximal femur decreased by 1.1%. In addition, studies on the effect of dietary supplementation (α-tocopherol 600 mg/die) on BMD, although few, are promising.

Further research is needed to better investigate the potential anabolic effect of vitamin E from food sources and from supplementation on bone.

## 4. Vitamin E and Loss of Muscle Mass and Power

This research has been carried out based on the keywords: “vitamin E” or “tocopherols” or “tocotrienols” AND “sarcopenia” AND “muscle mass”; 11 articles were sourced. Among them, one cross-sectional study, four reviews, two epidemiological study, two double-blind studies, and two animal studies have been selected and discussed.

Sarcopenia is defined by the European Working Group on Sarcopenia in Older People (EWGSOP) [42] as a syndrome characterized by progressive and generalized loss of muscle mass and strength. Sarcopenia is a physiological phenomenon that usually starts in the fifth decade. Van Kan has investigated the prevalence of sarcopenia in the population aged 60–70 years: In this age group, the prevalence ranged from 5% to 13%, but increased to 11%–50% in subjects aged >80 years [43]. Sarcopenia becomes responsible not only for the reduction of mobility and the level of autonomy of the elderly, but also for their ability to maintain good health. The functional reduction of the quadriceps muscle predisposes to a limitation in walking, with risk of falls and fractures of the femoral neck; a survey conducted in the USA has estimated the cost-related health consequences of sarcopenia to be 20–30 billion dollars [44]. In most elderly patients, the onset of sarcopenia is multifactorial and oxidative stress has been implicated as a central mechanism in the pathogenesis of sarcopenia [45]. Oxidative damage in skeletal muscle has been associated with the atrophy and loss of muscle function and fibers in sarcopenia [46]. Moreover, the accumulation of mitochondrial and nuclear DNA damage due to oxidative stress is thought to eventually compromise function, leading to the loss of myocytes [47]. Finally, reactive oxygen species can damage muscle tissue directly, but they also provide a trigger for the expression of inflammatory cytokines, such as interleukin- (IL-) 1, tumor necrosis factor (TNF), and IL-6. In older age, a low-grade inflammatory state characterized by increased concentrations of inflammatory cytokines and acute phase proteins is common and studies conducted among community-dwelling older adults suggest that the pro-inflammatory state does have a long-term consequence for loss of muscle mass and function [48,49]. Given this background, antioxidants, such as vitamin E, should play an important role against sarcopenia.

In particular, as regards vitamin E and loss of muscle mass and function, Semba *et al.* have been demonstrated that plasma higher α-tocopherol status were independently associated with higher strength measures [50] and frailty syndrome [51]. In this latest study, age- and gender-adjusted levels of vitamin E decreased gradually from the non-frail to the frail group. In the logistic model adjusted for multiple potential confounders, participants in the highest vitamin E tertile were less likely to be frail than were participants in the lowest vitamin E tertile (odds ratio, 0.30; 95% confidence interval, 0.10–0.91).

Interestingly, vitamin E plays a differential role in the oxidative metabolism in the various muscle fibers (types I and II). The type I fibers abound of myoglobin and mitochondrial enzymes and reconstitute phosphocreatine via oxidative phosphorylation more efficiently than the type II fibers [52], which theoretically generate more free radicals. Thus, it has been suggested that type I fibers (slow) require more vitamin E of type II fibers (fast) [52]. Furthermore, the high concentration of vitamin E has been associated with higher levels of activity of creatine kinase, suggesting the possibility of an increased repair of skeletal muscle [53]. An interesting longitudinal study evaluated the effect of concentrations of vitamin E and other various micronutrients (B6, B12, D, folic acid, iron) on the subsequent decline in physical function on a population of 698 people 65 and older belonging to a community [54]. The average decline according to the scale score Short Physical Performance Battery (SPPB) was 1.1. In logistic regression analysis, adjusted for potential confounders, only a low concentration of vitamin E (<1.1 µg/mL (<24.9 µmol/L)) was significantly associated with a subsequent decline in physical function (OR, 1.62; 95% confidence interval, 1:11 to 2:36; *p* = 0.01 for association of lowest levels of α-tocopherol quartile with at least a decrease of one point in physical function). In a general linear model, the concentration of vitamin E at the base, when analyzed as a continuous measure, was significantly associated with the SPPB score at follow-up, after adjustment for potential confounding factors, and score SPPB behind (β = 0.023; *p* = 0.01). According to a classification and regression analysis, age greater than 81 years and vitamin E (in participants aged 70–80 years) were identified as the most important determinant for the decline in physical function. The serum concentration of both α- and γ- tocopherol were associated with better physical function after hip fracture.

As regards vitamin E supplementation and loss of muscle mass and function, several studies have shown the positive effects of vitamin E supplementation in reversing muscle damage during extensive muscle contraction (exercise) in healthy men. Vitamin E supplementation at a dose of 800 IU for 28 days resulted in lowering the expression of oxidative stress markers after a downhill run in both young and older men [55].

In another study, a longer supplementation period (12 weeks of vitamin E supplementation) lowered creatinine kinase level after exercise in young men, whereas older men showed decreased lipid peroxidation in both resting state and after exercise, indicating that vitamin E promotes adaptation against exercise induced-oxidative stress and reduced muscle damage [56]. In animal models, similar results were obtained [57,58].

Table 3 summarizes the human studies to investigating the influence of vitamin E intake or supplementation on muscle mass.

In conclusion, to date epidemiological studies demonstrated that higher alpha-tocopherol status was associated with higher strength measures, so an adequate vitamin E intake avoids frailty syndrome in older subjects. Moreover, studies on vitamin E dietary supplementation (800 IU/die) demonstrated a decreased peroxidation and reduced muscle damage in both resting state and after exercise. However, no studies have evaluated the efficacy of a vitamin E intake or dietary supplementation in the elderly patient suffering from sarcopenia. These studies are needed, given that recent epidemiological studies in community-dwelling older adults show that low blood antioxidants are associated with low skeletal muscle strength and the development of walking disability and, consequently, frailty syndrome.

**Table 3:** Influence of vitamin E on muscle mass.

Author	Type of Study	Subjects Studied	Primary Endpoint	Main Results of the Study
Semba 2003 [50]	Cross-sectional analyses	669 non-disabled to severely disabled community-dwelling women aged 70 to 79 who participated in the Women’s Health and Aging Studies	To assess the association between dietary carotenoids and α-tocopherol with sarcopenia, as indicated by low grip, hip, and knee strength.	Higher carotenoid and α-tocopherol status were independently associated with higher strength measures.
Ble 2006 [51]	Epidemiological study	827 older (65 years) persons (women, 54%)	To evaluate the association between circulating levels of vitamin E and the presence of frailty.	Low plasma levels of Vit. E (α-tocopherol) are associated with frailty syndrome in older persons free from dementia and disability.
Bartali 2008 [54]	Longitudinal study	698 community-living persons (≥65 years)	To determine whether a low serum concentration of micronutrients (Vitamin E, B6, B12, Folic acid, D and Iron) are associated with subsequent decline in physical function among older men and women living in the community.	A low serum concentration of vitamin E is associated with subsequent decline in physical function among community-living older adults.
Dietary Supplementation				
Meydani 1993 [55]	Double-blind study	Nine young (22–29 years) and 12 older (55–74 years) sedentary male subjects	To measure the changes in oxidative products and antioxidants (α-tocopherol and γ-tocopherol) in skeletal muscle of young and old subjects after an eccentric exercise that causes delayed-onset muscle soreness and damage and then compare the antioxidant status of urine and plasma of older *vs.* younger.	All vitamin E-supplemented subjects excreted less (*p* < 0.05) urinary thiobarbituric acid adducts after the exercise bout than placebo subjects at 12 days post-exercise.
Sacheck 2003 [56]	Randomized clinical trial	Sixteen young (26.4 ± 3.3 years) and 16 older (71.1 ± 4.0 years) healthy men	To investigate the effects of an extended bout of downhill running on oxidative stress response and antioxidant status in healthy young and older men, and whether supplementation with vit, E could negate any observed differences.	Vitamin E supplementation (1000 IU α-tocopherol in soybean oil) induced modest changes eccentric exercise-induced oxidative stress, although differentially between the young and older subjects, while age had no direct influence on these responses among this group of physically fit subjects.

## 5. Vitamin E and Cognitive Performance

This research has been carried out based on the keywords: “vitamin E” or “tocopherols” or “tocotrienols” AND “mild cognitive impairment” AND “cognitive performance” AND “Alzheimer’s disease”; 24 articles were sourced. Among them one retrospective study, seven reviews, three case-control studies, four prospective studies, two animal studies, five observational studies, and two meta-analysis have been selected and discussed.

Epidemiological studies have reported a reduced incidence of dementia/Alzheimer’s disease (AD) in subjects with high plasma levels or high dietary intake of the combination of all natural vitamin E congeners [59,60,61], and *vice versa*, reduced plasma levels of α-tocopherol were found in subjects with AD, or mild cognitive impairment (MCI) [62,63,64,65].

Vitamin E is the major lipid-soluble, chain-breaking, non-enzymatic antioxidant in the human body [66], and is essential for normal neurological functions [67]. This micronutrient has thus been proposed as a preventive/therapeutic agent in AD, in which oxidative and nitrosative stress (OS/NS) promoted by free radicals seem to have a key pathogenetic role [68,69,70].

The same results have already demonstrated in animal models: at nanomolar concentrations, α-tocotrienol prevents neurodegeneration in mice and rat neurons, by regulating specific mediators of cell death [71].

However, until 2010 the role of different congeners of vitamin E mainly studied regarding cognitive performance was α-tocopherol [63,64,65] or all vitamin E congeners [59,60,61].

The few studies on cognition and plasma γ-tocopherol [72,73], and [74] δ-tocopherols yielded inconsistent results.

For the first time in a 2010 study, conducted in a population of Swedish octogenarians, a reduced incidence of AD was found in subjects with higher plasma levels of total tocopherols, total tocotrienols, and total vitamin E [60].

A 2012 study (Project-Add NeuroMed) confirmed these previous results: it was taken into account all vitamin E congeners in AD and MCI and comparing their plasma level with the relative markers of cognitive deterioration (α-tocopherylquinone, 5-nitro-γ-tocopherol) in 166 subjects with mild cognitive impairment, 168 patients with Alzheimer’s disease and 187 cognitively normal people [75]. The result was that low plasma levels of all vitamin E congeners have been associated with an increased likelihood of MCI and AD. Compared to cognitively normal subjects, AD and MCI had lower levels of total tocopherols, total tocotrienols and vitamin E total. In multivariate regression analysis-polytomic-logistics, both cases of MCI and cases of AD had an 85% lower chance of being in the highest tertile of total values of total tocopherols and vitamin E and they were, respectively, 92% and 94% less likely to be in the highest tertile of total tocotrienols compared to the lowest tertile [75].

In another study, a sample of 140 Finnish elderly people not suffering from cognitive disorders, come from the study Cardiovascular Risk Factors, Aging and Dementia (CAIDE), were followed over eight years to detect cognitive impairment, defined as the development of mild cognitive impairment (MCI) or Alzheimer's dementia. The results agree in pointing out that high levels of congeners of tocopherol and tocotrienol have been associated with a lower risk of cognitive impairment in the elderly [76]. All these results clearly testify the relevance of adequate vitamin E intake (15 mg/die is the dietary reference intake) in the economy of the antioxidant balance required to achieve healthy aging and preserve good cognitive performances.

The role of tocopherol could take a much more important therapeutically. Its use in mild form of Alzheimer’s disease could be a great help to slow its progression. This was highlighted in a randomized double-blind, placebo-controlled trial: in a group of patients it was found, after a median follow-up of two years, as the assumption of a fixed dose of tocopherol (2000 IU/die of α-tocopherol) has resulted in a better score ADCS-ADL Inventory, resulting in a delay in clinical progression of 19% per year compared with placebo or a delay of about 6.2 months in the period of follow-up [77].

As can be seen from the above studies, it emerges as vitamin E has an important role as an antioxidant against lipid peroxidation of cell membranes while preserving tissue cells from oxidative damage. Since vitamin E works both in the cytoplasm and nuclear, interacting with many genes related to inflammatory/immune response, it is important to know these correlations in order to create new frontiers for supplementation of vitamin E as correct as possible [78].

However α-tocopherol is still the only congener being tested in RCTs in subjects with AD or MCI, in doses up to 2000 IU/daily. Recent findings suggest that supplementation with high doses of α-tocopherol (800 UI/die) increased the risk for hemorrhagic stroke by 22% and reduced the risk of ischemic stroke by 10%; this differential risk pattern is obscured when looking at total stroke [79]. Further, α-tocopherol supplements can diminish the bioavailability of the other congeners: α-tocopherol supplementation can decrease plasma and tissue concentration of gamma- and δ-tocopherol [80], and compromise tissue delivery of tocotrienol [81].

It has been hypothesized that this contradictory result may be due to vitamin E-gene interactions; in particular polymorphisms of ApoE may be useful for vitamin E supplementation [78]. With regard to ApoE, ApoE4 genotype is associated with increased morbidity and mortality, and represents a significant risk factor for CVD cardiovascular disease and late-onset AD [82].

Table 4 summarizes the human studies performed to investigating the influence of vitamin E intake or supplementation on cognitive performance.

In conclusion, the results of the studies suggest that low serum levels and intake of tocopherols and tocotrienols are associated with the risk of cognitive impairment in older adults, which reinforces the hypothesis that each of the natural congeners of vitamin E plays a unique role in human health.

The complex and dynamic interactions between vitamin E and gene-interactions also need further clarification. Studies that take these factors into account can help determine the composition of vitamin E supplements for future AD prevention trials and refine dietary recommendations for healthy cognitive aging.

**Table 4:** Influence of vitamin E on cognitive performance.

Author	Type of Study	Subjects Studied	Primary Endpoint	Main Results of the Study
Devore 2010 [59]	prospective cohort study	5395 participants, aged 55+ years, who were free of dementia	To assess consumption of major dietary antioxidants in relation to long-term risk of dementia.	In multivariate models adjusted for age, education, APOE ε4 genotype, total energy intake, alcohol intake, smoking habits, body-mass index (BMI), and supplement use, higher intake of vitamin E at baseline was associated with a lower long-term risk of dementia (*p*-trend = 0.02). Higher intake of foods rich in vitamin E may modestly reduce long-term risk of dementia and AD.
Mangialasche 2010 [60]	Observational study	A dementia-free sample of 232 subjects aged 80+ years, derived from the Kungsholmen Project, was followed-up to 6 years to detect incident AD	To evaluate the association between plasma levels of eight congeners of vitamin E and incidence of Alzheimer’s disease (AD) among oldest-old individuals in a population-based setting.	The neuroprotective effect of vitamin E seems to be related to the combination of different congeners, rather than to α-tocopherol alone.
Morris 2005 [61]	Observational study	6158 community residents aged ≥65 years.	To assess the food intakes of vitamin E, α-tocopherol equivalents (a measure of the relative biologic activity of tocopherols and tocotrienols), or individual tocopherols protect against incident Alzheimer disease and cognitive decline over 6 y in participants of the Chicago Health and Aging Project.	Various tocopherol congeners rather than α-tocopherol alone may be important in the vitamin E protective association with Alzheimer disease.
Bourdel-Marchasson 2001 [62]	Case-control	20 patients with AD and 23 elderly control subjects living at home and free from disease	To investigate oxidative stress (plasma\erythrocyte malondialdehyde) and enzymatic and non-enzymatic antioxidants (α-tocopherol, retinol, GPx-peroxidase, superoxide dismutase) in normally nourished elderly with AD.	Lower plasma concentrations of α-tocopherol and retinol in normally nourished elderly patients with AD than in controls.
Perkins 1999 [63]	Cross-sectional	4809 non-Hispanic White, non-Hispanic Black, and Mexican-American elderly who visited the Mobile Examination Center during the Third National Health and Nutrition Examination Survey	To evaluate association between serum antioxidant (vitamins E, C, A, carotenoids, selenium) levels and poor memory performance in an elderly, multiethnic sample of the United States.	Decreasing serum levels of vitamin E per unit of cholesterol were consistently associated with increasing levels of poor memory after adjustment for age, education, income, vascular risk factors, and other trace elements and minerals. Serum levels of vitamins A and C, 3-carotene, and selenium were not associated with poor memory performance in this study.
Rinaldi 2003 [65]	Case-control	25 patients with MCI, 63 AD patients and 53 controls	To estimate peripheral levels and activities of Vitamin C, A, E, uric acid, carotenoids including lutein, zeaxanthin, α-cryptoxanthin, lycopene, α- and β-carotene as well as activities of plasma and red blood cell (RBC) superoxide dismutase (SOD) and of plasma glutathione peroxidase (GPx).	MCI patients showed significantly decreased plasma levels of vitamin C, E, A, uric acid, lutein, zeaxanthin, and α- carotene as compared to controls and significant lower activities of plasma and RBC SOD and of plasma GPx.
Kang 2008 [72]	Prospective study	858 female participants of the Nurses’ Health Study, aged 70+ years	To assess the association between plasma antioxidants (carotenoids and tocopherols) and cognition assessed by Telephone Interview for Cognitive Status-TICS (a telephone adaptation of MMSE), East Boston Memory test-EBMT, test of category fluency (name of animals in 1 min), digit span-back words.	In this population of generally well-nourished and healthy aging women, plasma carotenoid and tocopherol levels measured were not associated with their cognitive function or decline ten years later.
Schmidt 1998 [73]	Evaluation of cross-sectional data from a cohort study	A total of 1769 subjects aged 50 to 75 years, with no history or signs of neuropsychiatric disease, selected randomly from the community register	To evaluate the association between cognitive status and plasma concentrations of various antioxidants (lutein/zeaxanthin, cryptoxanthin, canthaxanthin, lycopene, alpha-carotene, beta-carotene, retinol, gamma-tocopherol, alpha-tocopherol) in middle-aged and older individuals without neuropsychiatric disease.	Only alpha-tocopherol remained significantly associated with cognitive functioning when logistic regression analysis was used to adjust for possible confounders including age, sex, month of blood sampling, years of education, smoking, lipid status, and major risk factors for stroke.
Ravaglia 2008 [74]	Cohort study	761 elderly Italian subjects from a population based cohort assessed in 1999–2000 for mild cognitive impairment (MCI) and dementia. In 2003–2004, information about cognitive status was collected for 615 of the 666 subjects without baseline cognitive impairment	To investigate plasma concentrations of the natural tocopherols and the tocopherol oxidation markers α-tocopherylquinone (αTQ) and 5-nitro-α-tocopherol (5NGT) in relation to cognitive function in the elderly.	Plasma concentrations of some non-α-tocopherol congeners of vitamin E are associated with cognitive impairment in elderly people. However, the associations depend on concurrent cholesterol concentration and need further investigation.
Mangialasche 2012 [75]	Observational study	168 AD cases, 166 MCI, and 187 cognitively normal (CN) people	To assess the relation of all plasma vitamin E congeners and markers of vitamin E damage (α-tocopherylquinone, 5-nitro-γ-tocopherol) with mild cognitive impairment (MCI) and Alzheimer’s disease (AD).	Both disorders were associated with increased vitamin E damage. Low plasma tocopherols and tocotrienols levels are associated with increased odds of MCI and AD.
Mangialasche 2013 [76]	Longitudinal retrospective case-control study	140 non-cognitively impaired elderly	To investigate the association between serum levels of tocopherols and tocotrienols, markers of vitamin E oxidative/nitrosative damage (α-tocopherylquinone, 5-nitro-γ-tocopherol) and incidence of cognitive impairment.	Elevated levels of tocopherol and tocotrienol congeners are associated with reduced risk of cognitive impairment in older adults. The association is modulated by concurrent cholesterol concentration.
**Dietary Supplementation**				
Dysken 2014 [77]	Double-blind, placebo-controlled, parallel-group, randomized clinical trial	613 patients with mild to moderate AD	To determine if supplementation with vitamin E (2000 IU/d of α-tocopherol), memantine (20 mg/die), or both slow progression of mild to moderate AD in patients taking an acetylcholinesterase inhibitor.	Among patients with mild to moderate AD, 2000 IU/d of α-tocopherol compared with placebo resulted in slower functional decline. There were no significant differences in the groups receiving memantine alone or memantine plus α-tocopherol.

## 6. Discussion

All studies performed in order to assess vitamin E intake or serum levels of tocopherols and tocotrienols in elderly population are in agreement: older subjects are at high risk of vitamin E deficiency [4,5,6,8].

Intake of α-tocopherol below 12 mg/d has been shown to relate to risk of hydrogen peroxide-induced hemolysis [10], and there is evidence of association in adult population with several major chronic diseases, including diabetes, cancer, and cardiovascular disease [11,12,13,14].

In addition to the chronic diseases associated with a deficiency of vitamin E intake demonstrated in adults, in the elderly, the deficit of vitamin E is associated with diseases closely linked to aging, such as osteoporosis, sarcopenia, and cognitive deficits.

On the contrary, both high intake and serum concentrations of α-tocopherol have been shown to be associated with reduced risk of many common sequelae of hip fracture, including decreased physical function, incident frailty, sarcopenia, bone loss, and cognitive decline. Higher intake with diet (15 mg/die) or vitamin E supplementation (600 UI/die) may represent a potentially modifiable factor for preventing loss of bone and muscle mass and cognitive decline.

Therefore, it is important to make known the elderly regarding which foods contain the vitamin compounds of the group E in order to make richer their nutrition in this vitamin. Vitamin E is found in many plants, especially in the seeds, oil derivatives, grains, fruits, and vegetables [83], as shown in Table 5, Table 6 and Table 7. Another significant element is the type of style food taken by the elderly subjects; in fact, people who use rice as the main cereals should choose the type of cultivars most rich in vitamin E. The result obtained from a study of 58 rice varieties cultivated in Malaysia was that vitamin E can range from 19:36 to 63.29 mg/kg, and the data particularly interesting is that all varieties of rice contain more isomers of tocotrienol than tocopherol [84]. This could address the choice depending on the cultivar richest in bioactive compounds.

Regarding vitamin E supplementation, however, meta-analyses evaluating the antioxidant effect of vitamin E have reported a disappointing effect on survival [85,86,87,88,89]. Among these meta-studies, Miller demonstrated that low-dose vitamin E slightly, but non-significantly, decreased the mortality, but high-dose vitamin E increased mortality [87]. High dosage of vitamin E intake has been revealed to contribute to many clinical disorders [90], which is consistent with the increased mortality for high-dose vitamin E. Otherwise, another meta-study on vitamin E, which used the same inclusion criteria as Miller *et al.* [87], but adopted a different meta-analytic method, also got a disappointing result [91,92]. Such an effect could obscure the effect of vitamin E in studies without considering the dosage, which is seen in several reports [85,86,88,89]. The cooperative effect of vitamin E with other agents could also be concealed in studies pooling vitamin E alone and with other agents together, which is seen in several reports [85,86,87,89,91]. In any case, the meta-analysis conducted to date have not specifically considered the age of subjects studied and different congeners of vitamin E.

**Table 5:** Content of vitamin E, α-tocopherol eq., in some foods [93].

Food	mg/100 g	Food	mg/100g
Wheat germ oil	136.70	tomato paste	5.37
sunflower oil	49.20	pistachios	5.21
seed oil corn	34.50	sticks and crackers integral	3.92
extra virgin olive oil	21.42	muesli	3.20
Hazelnut	24.98	hen egg, yolk	3.11
Sweet almond	23.96	chickpeas (dry)	2.61
peanuts (not roasted)	10.09	butter	2.40
Caviar	7	chicory	2.26
Eel river	5.55	low-fat milk	0.04

In elderly populations it is relevant to consider the different congeners of vitamin E supplementation for its possible beneficial effect on the entire health in aging taking into account that vitamin E also affects the inflammatory/immune response [22]. The current formulation of vitamin E consists primarily of α-tocopherol, but recent research has suggested that tocotrienol, the lesser known congener of vitamin E, appears superior regarding its antioxidant properties [94] and possesses unique biological functions unrelated to antioxidant activity not shared by tocopherol [95].

**Table 6:** Content of vitamin E in mg/100 g in selected food samples (Karmowski, J.; *et al.* [83]).

Vitamin E mg/100 g	Corn Oil	Peanut Oil	Sesame Oil	Sunflower Oil	Walnut Oil	Milk	Milk Cream
α-tocopherol	13 ± 0.3	20 ± 1	4 ± 0.2	46 ± 4	8 ± 1	0.07 ± 0.01	0.5 ± 0.04
β-tocopherol	-	-	-	-	-	-	0.05 ± 0.004
γ-tocopherol	87 ± 8	15 ± 0.6	0.3 ± 0.02	-	29 ± 0.3	-	-
γ-tocopherol	-	-	-	-	5.0 ± 0.6	-	-
α-tocotrienol	-	-	-	-	-	-	-
β-tocotrienol	-	-	-	-	-	-	-
γ-tocotrienol	-	-	-	-	-	-	-
δ-tocotrienol	-	-	30 ± 7	-	-	-	-

**Table 7:** Content of vitamin E in mg/100 g in selected cereals (Karmowski, J.; *et al.* [83]).

Vitamin E mg/100 g	Badengold	Luteus	Kombo	Macaroni	Rotkorn	Tommi
α-tocopherol	0.02 ± 0.002	0.04 ± 0.003	0.07 ± 0.01	0.02 ± 0.003	0.03 ± 0.01	0.05 ± 0.01
β-tocopherol	0.3 ± 0.02	-	0.2 ± 0.01	0.06 ± 0.002	0.25 ± 0.01	0.3 ± 0.1
γ-tocophero	-	-	-	-	-	-
δ-tocopherol	-	0.01 ± 0.001	-	-	-	-
α-tocotrienol	<LOQ	<LOQ	0.1 ± 0.01	0.01 ± 0.003	<LOQ	0.03 ± 0.01
β-tocotrienol	1.7 ± 0.1	1.2 ± 0.1	2.9 ± 0.01	0.9 ± 0.04	1.6 ± 0.1	2.3 ± 0.3
γ-tocotrienol	0.01 ± 0.001	0.01 ± 0.001	0.02 ± 0.001	-	<LOQ	<LOQ
δ-tocotrienol	-	0.01 ± 0.001	<LOQ	-	-	<LOQ

LOQ: limit of quantification.

Even among the tocopherols, particular importance is placed on the other isomers because supplementation with large doses of α-tocopherol alone has been reported to deplete the availability of γ-tocopherol, thus denying the benefits of α-tocopherol that are not shared by γ-tocopherol [96]. Therefore, it has been suggested that the full benefits of vitamin E are better achieved in the elderly by supplementation with the full spectrum of vitamin E isomers and the corresponding tocotrienols [97,98].

## 7. Conclusions

In conclusion, the recent literature underlines that, given the detrimental effect of low intake and serum levels of tocopherols and tocotrienols on bone, muscle mass, and cognitive function, a change in the lifestyle must be the cornerstone in the prevention of these specific age-related pathologies related to vitamin E deficient status. The optimum diet therapy in elderly for avoiding vitamin E deficiency intake and its negative correlates, such as high inflammation and oxidation which are the cause of most age-related pathologies, such as osteoporosis, sarcopenia, and cognitive impairment, must aim at achieving specific nutritional goals. These goals must be reached through accession of the elderly subjects to specific personalized dietary program aimed at achieving and/or maintaining body weight (avoid malnutrition); increase their intake of all food rich in vitamin E, such as derivatives oily seeds (in particular wheat germ oil), olive oil, nuts (hazelnuts, walnuts, almonds), and cereals rich in vitamin E (such as specific rice cultivar rich in tochotrienols) or take vitamin E supplements. Vitamin E supplementation can be correctly used in a personalized way either for the outcome from the pathology or to achieve healthy aging and longevity without any adverse effects.
